# Preconception Stress and Pregnancy Serum Glucose Levels Among Women Attending a Fertility Center

**DOI:** 10.1210/jendso/bvad152

**Published:** 2024-01-04

**Authors:** Lidia Mínguez-Alarcón, Olivia Chagnon, Aya Tanaka, Paige L Williams, Tamarra James-Todd, Jennifer B Ford, Irene Souter, Kathryn M Rexrode, Russ Hauser, Jorge E Chavarro

**Affiliations:** Channing Division of Network Medicine, Harvard Medical School & Brigham and Women's Hospital, Boston 02115, USA; Department of Environmental Health, Harvard T.H. Chan School of Public Health, Boston 02115, USA; Department of Environmental Health, Harvard T.H. Chan School of Public Health, Boston 02115, USA; Department of Epidemiology, Harvard T.H. Chan School of Public Health, Boston 02115, USA; Department of Epidemiology, Harvard T.H. Chan School of Public Health, Boston 02115, USA; Department of Biostatistics, Harvard T.H. Chan School of Public Health, Boston 02115, USA; Department of Environmental Health, Harvard T.H. Chan School of Public Health, Boston 02115, USA; Department of Epidemiology, Harvard T.H. Chan School of Public Health, Boston 02115, USA; Department of Environmental Health, Harvard T.H. Chan School of Public Health, Boston 02115, USA; Division of Reproductive Medicine and IVF, Department of Obstetrics and Gynecology, Massachusetts General Hospital and Harvard Medical School, Boston, MA 02114, USA; Division of Women's Health, Department of Medicine, Brigham and Women's Hospital, Harvard Medical School, Boston, MA 02115, USA; Department of Environmental Health, Harvard T.H. Chan School of Public Health, Boston 02115, USA; Department of Epidemiology, Harvard T.H. Chan School of Public Health, Boston 02115, USA; Department of Obstetrics, Gynecology and Reproductive Biology, Harvard Medical School, Boston 02115, USA; Channing Division of Network Medicine, Harvard Medical School & Brigham and Women's Hospital, Boston 02115, USA; Department of Epidemiology, Harvard T.H. Chan School of Public Health, Boston 02115, USA; Department of Nutrition, Harvard T.H. Chan School of Public Health, Boston, USA

**Keywords:** glucose levels, impaired glucose tolerance, perceived stress, subfertility

## Abstract

**Context:**

The association between women's stress and pregnancy glucose levels remain unclear, specifically when considering the preconception period as a sensitive window of exposure.

**Objective:**

We investigated whether preconception perceived stress was associated with glucose levels during pregnancy among women attending a fertility center (2004-2019).

**Methods:**

Before conception, women completed a psychological stress survey using the short version of the validated Perceived Stress Scale 4 (PSS-4), and blood glucose was measured using a 50-gram glucose load test during late pregnancy as a part of screening for gestational diabetes. Linear and log-binomial regression models were used to assess associations of total PSS-4 scores with mean glucose levels and abnormal glucose levels ( ≥ 140 mg/dL), adjusting for age, body mass index, race, smoking, education, physical activity, primary infertility diagnosis, number of babies, and mode of conception.

**Results:**

Psychological stress was positively associated with mean abnormal glucose levels. The adjusted marginal means (95% CI) of mean glucose levels for women in the first, second, and third tertiles of psychological stress were 115 (110, 119), 119 (115, 123), and 124 (119, 128), and mg/dL, respectively (*P* for trend = .007). Also, women in the second and third tertiles of psychological stress had 4% and 13% higher probabilities of having abnormal glucose compared with women in the first tertile of psychological stress (*P* trend = .01).

**Conclusion:**

These results highlight the importance of considering preconception when evaluating the relationship between women's stress and pregnancy glucose levels.

Stress prevalence has increased over the years. Among 2281 participants in the National Study of Daily Experiences showed, higher levels of stress were observed in the 2010s compared with the 1990s [1, 2]. In recent years, high levels of stress were observed in a 2020 survey including 1523 respondents from 48 countries, potentially due to the COVID-19 pandemic [3], and other studies have concluded similar findings [4, 5]. Epidemiologic research has also shown that gender differences are prevalent in perceived stress, with women experiencing higher levels of self-reported stress than men [6-10]. Special attention has been given to women conceiving through assisted reproductive techniques as women with fertility problems have an elevated risk of adverse metabolic conditions, such as polycystic ovarian syndrome (PCOS). Likewise, metabolic responses following assisted reproductive technologies may be linked to glucose dysregulation in pregnancy [11], making individuals with reduced fertility a key target study population to evaluate predictors of gestational diabetes mellitus (GDM) and impaired glucose during pregnancy. Interestingly, subfertile women also experience higher rates of psychological stress as well as anxiety, depression, and poor quality of life [12].

Studies in pregnant animals have demonstrated that stress can affect glucose metabolism. For example, pregnant female rats subjected to stressors (single housing and saline injection) had altered hepatic glucogenic capacity [13]. In a study of 28 pregnant ewes, increased maternal cortisol, a biomarker of stress, was associated with an increased maternal glucose concentration [14]. Human studies evaluating stress in relation to glucose levels during pregnancy have mostly focused on assessing stress during pregnancy and results have been mixed [15-20]. Epidemiologic evidence has shown that lifestyle factors and environmental stressors assessed within the preconception window may be important predictors of maternal health during pregnancy. Specifically, human studies have found that preconception diet [21-26], physical activity [27], and exposures to certain air pollutants [28] may be associated with GDM. Studies have also found that preconception physical activity [29, 30] may be associated with preeclampsia and that preconception nutritional factors may also be associated with hypertensive disorders of pregnancy [31]. In addition, it is unclear whether the relationship of maternal perceived stress with pregnancy glucose levels differs by socioeconomic status. This is particularly important, as stress has been related to socioeconomic factors often affecting health [32, 33]. For example, income and education were more strongly associated with stress in Black adults compared with White adults [34]. Here, we investigated whether maternal stress, evaluated during the preconception period, was associated with blood glucose levels during pregnancy among women attending a fertility center. We also evaluated whether the associations varied by mode of conception (natural, intrauterine insemination [IUI] and in vitro fertilization [IVF]) and selected socioeconomic factors (race, education, income).

## Subjects and Methods

### Study Population

This prospective analysis included women who enrolled in the Environment and Reproductive Health (EARTH) Study, a prospective cohort established to assess environmental and dietary determinants of fertility at the Massachusetts General Hospital Fertility Center [35]. Between 2004 and 2019, 1324 women between 18 and 45 years of age who were seeking fertility care at the center were eligible to participate, and 991 of those contacted by the research staff enrolled prior to conception in the study (recruitment is not ongoing since the study already ended). This analysis includes 398 women who self-reported preconception perceived stress at study entry and had glucose levels measured during pregnancy. The study was approved by the Human Subject Committees of the Harvard T.H. Chan School of Public Health and Massachusetts General Hospital. Participants signed an informed consent after the study procedures were explained by trained research study staff and all their questions were answered.

### Self-Reported Perceived Stress Assessment

We evaluated perceived stress using the short form of the Perceived Stress Scale (PSS-4) [36]. Women were specifically asked during the past 3 months: “How often have you felt that you were unable to control the important things in your life?”; “How often have you felt confident about your ability to handle your personal problems?”; “How often have you felt that things were going your way?”; and “How often have you felt difficulties were piling up so high that you could not overcome them?” Responses included never, almost never, sometimes, fairly often, and very often. Self-reported perceived stress is assessed as the total scores of each item ranging from 0 (lowest score/stress) to 16 (highest score/stress). As the PSS-4 is not a diagnostic tool, there are no reference values to categorize stress level in examined subjects. Thus, we used the total score as a continuous exposure as well as a categorical variable divided in approximate tertiles (T1 = 0-3, T2 = 4-6, T3 = 7-14; lowest tertile was the reference group) based on the distribution among women in this study in main analyses. Within the stratified analyses, we used perceived stress as a continuous exposure to increase study power. The validity of the PSS-4 to evaluate psychological stress has been previously confirmed when compared with other validated depression and anxiety instruments among 37 451 European subjects [37] and in other smaller studies [38, 39]. Furthermore, the PSS-4 (short version) score has demonstrated high correlation with the PSS-10 (long version) (*r* = 0.91) and similar correlations as the PSS-10 with depressive symptoms (*r* = 0.41 and *r* = 0.46, respectively) among Mexican women [40].

### Pregnancy Glucose Level Assessment After Glucose Load Testing

Blood glucose levels during pregnancy were assessed using a 1-hour nonfasting, 50-g glucose load test (GLT) at late-second/early-third trimester as part of the two-step method using the Carpenter-Coustan criteria for GDM screening [41] among women in this study. Data were abstracted from medical records. We used pregnancy blood glucose levels as a continuous outcome and also as binary outcome (normal vs abnormal glucose levels) defined as pregnancy glucose ≥ 140 mg/dL [42], in which women would have been referred for additional GDM screening based on the elevated glucose level.

### Covariate Assessment

The participant's date of birth was collected at entry, and weight and height were measured by trained study staff. Body mass index (BMI) was calculated as weight (in kilograms) divided by height (in meters) squared. At enrollment, research staff administered sociodemographic, lifestyle, and medical history questionnaires to participants. Study participants also completed a comprehensive questionnaire on family, medical, reproductive, and occupational history, perceived stress, consumer products use, smoking history, and physical activity. Total physical activity was calculated as the sum of vigorous, moderate, and light self-reported leisure exercise. The median family income (in 2011 inflation-adjusted dollars) in the past 12 months from the American Community Survey 2007-2010 within the census tract of a participant's preconception residential address was used as a proxy for the socioeconomic status of individual participants. Infertility was diagnosed using the Society of Assisted Reproductive Technology definitions. Information on mode of conception (natural, IUI with or without ovulation induction, and IVF with or without intracytoplasmic sperm injection [ICSI]), and infant sex as recorded at birth, was abstracted from medical records.

### Statistical Analysis

We present maternal and child demographic characteristics as well as reproductive characteristics using median ± interquartile ranges (IQRs) or percentages. We used adjusted linear regression models to evaluate the relationships of self-reported preconception psychological stress with continuous blood glucose levels and presented results as estimated betas (95% CI), representing the mean difference in blood glucose (mg/dL). We used adjusted log-binomial models to estimate the association between self-reported perceived stress and probabilities of abnormal pregnancy glucose levels and presented the results as risk ratios (RR) (95% CI). To allow for better interpretation of the results when using preconception maternal stress as a categorical variable (tertiles), we translated model estimates to obtain estimated population marginal means. Confounding was assessed using both prior knowledge regarding biological relevance and descriptive statistics from our study population. Variables related with both psychological stress levels and the outcomes that were not in the causal pathway were considered as confounders [43, 44]. Adjusted models included at enrollment's age (years), BMI (kg/m^2^), race (White and Black/Asian/other), smoking status (current and ever/never smoked), physical activity (hours/week), education (college degree and other), primary infertility diagnosis (female factor and male/unexplained), multifetal gestation (yes and no) and mode of conception (natural, IUI/ovulation induction, and IVF including ICSI). We also evaluated potential effect modification by conducting stratified analyses by mode of conception (natural, IUI, and IVF) and selected socioeconomic indicators including race (White and Black/Asian/other), education (college degree and other) and census-tract median annual household income (< $100 000 and ≥ $100 000). Statistical analyses were performed with SAS (version 9.4; SAS Institute Inc., Cary, NC, USA).

## Results

Women in this analysis had a median (IQR) BMI of 23.4 kg/m^2^ (21.3, 26.4) and age of 35 years (32, 38) at study entry. Most women were of White ethnic background (83%), reported never smoking (78%), and were generally highly educated (64% with at least a college degree) (Table 1). Three hundred (75%) women got pregnant using medically assisted technologies (65 women [16%] using IUI and 235 [59%] using IVF). Women had a median (IQR) PSS-4 score of 5 (3, 7), with a range of 0 to 14. Median (IQR) pregnancy blood glucose levels (mg/dL) were 119 (98, 135) post glucose load. Glucose testing was done at a median of 26 weeks (IQR = 25-30) of pregnancy and glucose measures were taken at 1 hour after a 50 g glucose load. The mean glucose level was 119 mg/dL (IQR 98-135). A total of 82 women (21%) had abnormal gestational glucose levels (≥140 mg/dL) post glucose load (Table 1). Compared with women included in this analysis, those who were excluded had similar demographic and reproductive characteristics, as well as similar PSS-4 scores, although the excluded women were slightly less educated. Women with abnormal gestational glucose had a mean of 6 PSS-4 scores and women without abnormal glucose levels had a mean of 5 PSS-4 scores. The median (IQR) elapsed time between assessment of stress and blood glucose assessment was 314 (219, 476) days.

**Table 1.** Demographic and reproductive characteristics shown as median (IQR or N (%) among included and excluded women from the Environment and Reproductive Health (EARTH) Study

	Included (N = 398)	Excluded (N = 593)
Age, years	35.0 (32.0, 38.0)	35.0 (32.0, 39.0)
Race, N (%)		
White	331 (83)	498 (84)
Black	13 (4)	24 (4)
Asian	37 (9)	47 (8)
Other	17 (4)	24 (4)
Body mass index, kg/m^2^	23.4 (21.3, 26.4)	23.2 (21.6, 26.6)
Smoking, N (%)		
Never smoker	310 (78)	310 (78)
Past smoker	80 (20)	80 (20)
Current smoker	8 (2)	8 (2)
Total physical activity, hours/wk	5.45 (2.70, 9.68)	5.50 (2.50, 9.93)
Education, N (%)		
High school or less	18 (5)	59 (10)
College	124 (31)	184 (33)
Graduate degree	256 (64)	332 (56)
Census-tract median income*^a^*, $	103 000 (76 700, 134 000)	96 300 (77 600, 123 000)
Total PSS-4 scores, n	5 (3, 7)	5 (3, 8)
Mode of conception, N (%)		
Natural	98 (25)	119 (20)
IUI	65 (16)	83 (14)
IVF/ICSI	235 (59)	391 (66)
Initial infertility diagnosis, N (%)		
Male factor	97 (24)	148 (25)
Female factor	116 (29)	190 (32)
Unexplained	185 (47)	255 (43)
Pregnancy glucose levels, mg/dL	119 (98.0, 135)	—
Abnormal glucose levels*^b^*, N (%)	82 (21)	—

Abbreviations: ICSI, intracytoplasmic sperm injection; IUI, intrauterine insemination; IVF, in vitro fertilization; PSS-4, Perceived Stress Scale.

^*a*^N = 209 women.

^*b*^Defined as glucose levels ≥ 140 mg/dL.

We found that women with higher preconception PSS-4 scores had higher mean glucose levels in both unadjusted and adjusted models (Table 2). Similarly, we observed positive associations between stress in tertiles and mean glucose levels. Specifically, the adjusted marginal means (95% CI) of mean glucose levels for women in the lowest, middle, and highest tertiles of self-reported perceived stress were 115 (110, 119), 119 (115, 123), and 124 (119, 128), and respectively (*P* for trend = .007). In addition, stress, assessed continuously and in tertiles, was positively related to the probability of having abnormal GLT glucose levels among women in this study. Specifically, women in the middle and highest tertiles of perceived stress had 4% and 13% higher probabilities of having abnormal glucose levels compared with women in the lowest tertile of self-reported perceived stress (*P* for trend = .01) (Table 2).

**Table 2.** Pregnancy glucose levels by preconception perceived stress among 398 women in the Environment and Reproductive Health (EARTH) Study

	Unadjusted glucose levels, mg/dL	Adjusted*^a^* glucose levels, mg/dL	Unadjusted abnormal glucose levels, estimated probability	Adjusted*^a^* abnormal glucose levels, estimated probability
Continuous PSS-4 scores β estimate (95% CI)*^b^*	0.90 (0.04, 1.77)	0.83 (−0.02, 1.69)	1.08 (1.00, 1.17)	1.08 (0.99, 1.17)
*P* value	0.04	0.05	0.06	0.08
Categorical PSS-4 scores Predicted marginal means (95% CI)				
Lowest: T1 [0-3]	115 (110, 119)	115 (111, 120)	0.15 (0.10, 0.22)	0.14 (0.09, 0.21)
Middle: T2 [4-6]	119 (115, 123)	119 (115, 123)	0.20 (0.14, 0.27)^†^	0.18 (0.13, 0.26)^†^
Highest: T3 [7-14]	124 (119, 129)*	124 (119, 128)*	0.28 (0.21, 0.36)*	0.27 (0.19, 0.36)*
*P* trend	0.007	0.009	0.01	0.01

Abbreviation: PSS-4, Perceived Stress Scale.

^*a*^Models are adjusted for age, BMI, race, smoking, education, physical activity, primary infertility diagnosis, number of babies delivered, and mode of conception.

^*b*^RR (95% CI) for impaired glucose levels (binary outcome).

^*^ *P* value <.05 when compared that tertile with the lowest tertile of exposure.

^†^ *P* value <.10 when compared that tertile with the lowest tertile of exposure.

**Figure 1.**
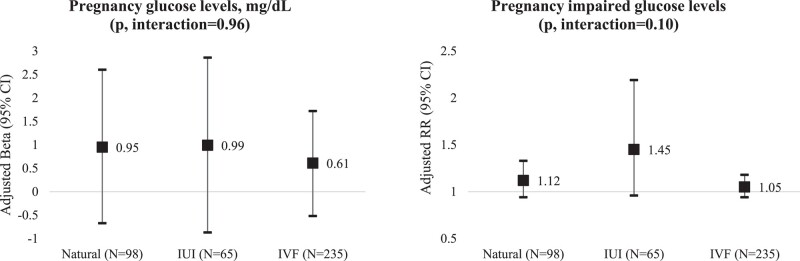
Adjusted pregnancy glucose levels by preconception perceived tress stratified by mode of conception among 398 women in the Environment and Reproductive Health (EARTH) study. Models are adjusted for age, BMI, race, smoking, education, physical activity, primary infertility diagnosis, and multifetal gestation.

In analyses stratified by mode of conception, the positive association between stress and abnormal glucose was stronger among women who conceived using IUI (RR [95% CI] = 1.45 [1.96, 2.19]); however, lack of study power was a concern given the sample size in these analyses (N = 65) (Fig. 1). Also, preconception perceived stress was more positively related to mean blood glucose levels among women with a college degree (β [95% CI] = 1.24 [0.16, 2.32]) than among women with lower education levels (β [95% CI] = 0.01 [−1.38, 1.39] mg/dL), and among women with higher income (β [95% CI] = 1.76 [0.46, 3.07] mg/dL) compared to those with lower income (β [95% CI]= −0.09 [−2.22, 2.04] mg/dL) (Table 3). We did not find any other associations between preconception self-reported psychological stress with mean or abnormal pregnancy glucose levels in stratified analyses by other socioeconomic factors.

**Table 3.** Adjusted^**a**^ pregnancy glucose levels by preconception perceived stress stratified by certain social determinants among 398 women in the Environment and Reproductive Health (EARTH) Study

	Race		Education		Income, $	
	White	Black, Asian, Other	Undergrad or less	College degree	<100 000	>=100 000
Continuous glucose levels β estimate (95% CI)	0.87 (−0.06, 1.80)	0.30 (−1.89, 2.48)	0.01 (−1.38, 1.39)	1.24 (0.16, 2.32)	−0.09 (−2.22, 2.04)	1.76 (0.46, 3.07)
Abnormal glucose levels RR (95% CI)	1.14 (0.96, 1.34)	1.11 (0.93, 1.33)	0.98 (0.85, 1.15)	1.12 (1.01, 1.24)	0.99 (0.78, 1.25)	1.13 (0.96, 1.34)

Note: *P* for interactions of race and stress with continuous and abnormal glucose were .72 and .50, respectively. *P* for interactions of education and stress with continuous and abnormal glucose were .23 and .29, respectively. *P* for interactions of income and stress with continuous and abnormal glucose were .05 and .18, respectively.

^*a*^Models are adjusted for age, BMI, race, smoking, education, physical activity, primary infertility diagnosis, multifetal gestation, and mode of conception.

## Discussion

Among women participating in the EARTH Study, we examined the associations of self-reported perceived stress using the PSS-4 scale during preconception with 1-hour blood glucose levels after 50 g glucose load test during pregnancy. We also explored whether this relationship was modified by mode of conception and also by different groups of socioeconomic factors. We found that higher preconception psychological stress was associated with higher pregnancy blood glucose levels as well as the probability of having abnormal glucose levels during pregnancy. The positive association between stress and abnormal glucose was particularly stronger among women conceiving using IUI. We also observed that the positive association between preconception perceived stress and GLT glucose levels was only observed among women with a college degree as well as those with a census-tract median annual household income >$100 000. These results highlight the importance of considering preconception as a sensitive window of stress in relation to cardiovascular health during pregnancy.

Mean total PSS-4 scores among women in this study were similar to other reported studies in pregnant women in Spain (mean = 5.43) [37] and France (mean = 5.40) [45]. However, participants in China (mean ∼6) [46] and Korea (mean ∼8) [47] reported higher mean PSS-4 scores.

Most of the epidemiologic literature on perceived stress and glucose levels during pregnancy has only evaluated gestational stress, as studies did not consider the preconception window. For example, in the New York State Pregnancy Risk Assessment Monitoring System (PRAMS) Survey (2004-2006) among 2690 pregnant women, authors found that having 5 or more stressful events (reported without using any validated instrument) in the 12 months before the baby was born was significantly associated with GDM [15]. Maternal stress, assessed using the state-trait anxiety inventory (STAI) trait and state questionnaires for stress assessment during the second and third trimesters of pregnancy, was associated with decreased insulin sensitivity in 82 pregnant women recruited in an obstetrics and gynecology outpatient clinic of a university hospital in Greece (2015-2016) [16]. Maternal prenatal perceived stress (measured using the PSS-10 questionnaire) was positively related to odds of GDM in a case control study including 100 cases at 20+ weeks gestation and 273 matched controls at 2 hospitals in India (2014-2016) [17]. However, authors found no association between perceived stress during pregnancy and maternal blood glucose levels. Among 203 pregnant women recruited at a Swiss university hospital (2012-2013), authors found that number of pregnancy-related major life events was significantly associated with fasting glucose levels measured at 24 to 30 weeks of gestation [18]. Perceived stress within the last month, measured using the PSS-14 at 24 to 30 weeks of gestation, was unrelated to fasting glucose concentrations. Additionally, higher levels of general distress, measured by the DASS-21 total score, and shorter self-reported sleep durations were independently associated with higher fasting glucose levels; however, these results were not significant after controlling for age and BMI. Among 832 pregnant women in early and mid-pregnancy recruited at the University of Colorado Anschutz Medical Center (2010-2014), authors found that mental health status, including perceived stress and depression measured by the PSS-10 and Edinburgh Postnatal Depression Scale, respectively, was not individually associated with risk of dysglycemia [19]. Results of the study found that having multiple positive modifiable factors during pregnancy, including a healthy diet, high physical activity level, and lower stress level, was associated with a reduced risk of prenatal dysglycemia. Perceived stress, measured using the PSS-14 both in early and mid-pregnancy, was not associated with glucose intolerance during pregnancy among 1115 Hispanic (predominantly Puerto Rican) women participating in Proyecto Buena Salud (2006-2011) [20]. However, increased stress from early to mid-pregnancy was related to glucose intolerance. Discrepancies in results across all these studies, which evaluated stress during pregnancy in relation to GDM or gestational glucose levels, may be due to differences in instruments to collect psychological stress, study designs, racial/ethnic background, and other important demographics associated with stress. Also, differences in gestational age and glucose test may contribute to these differences across studies.

We observed that stress was more strongly associated with impaired glucose among women following IUI. This may be explained by the fact that IUI treatment has shown less effectiveness as an infertility treatment compared to IVF, so women undergoing IUI may experience more distress compared to IVF women. Specifically in the EARTH Study, IVF cycles resulted in more live births compared to IUI. In line with this hypothesis, perceived stress was positively related to female factor infertility among 286 women and 236 men seeking to become parents through fertility treatment in Canada [48] and female factor infertility is more prevalent in IUI cycles because IVF was traditionally the preferred treatment for couples with male factor infertility [49]. In fact, male factor infertility was more prevalent among IVF cycles compared to IUI in our study. One possible explanation for the stronger associations seen in women with higher incomes and attained education levels is that many of these individuals may be employed in demanding, time-intensive jobs. It has previously been shown that those with a higher education level experience greater levels of job stress, with stronger associations found in women than in men [50]. Given that education level is positively associated with salary [51], it is possible that this explanation applies to women with higher incomes as well. Professional women are often also responsible for balancing demands in the workplace with household duties and childcare, which can increase levels of stress in the zona fasciculata of the adrenal cortex [52-54].

Stress is known to initiate a physiological response that results in the activation of the hypothalamic-pituitary-adrenal (HPA) axis, which stimulates the production of glucocorticoids in the adrenal cortex [55-57]. The presence of glucocorticoids, including cortisol, limits the transport of glucose into skeletal muscle and adipose tissue and stimulates the gluconeogenesis pathway in the liver [14, 18, 58, 59]. While the resulting high blood glucose level ensures that there is adequate energy supply during the fight or flight response, repeated activation of the HPA under chronic stress can lead to insulin resistance and hyperglycemia [60-63].

Our study has important limitations. First, the generalizability of the observed results to women in the general population is of concern as this study comprises a group of women seeking fertility care, which can be stressful. However, this also provides a unique opportunity to evaluate stratification by mode of conception and evaluate a group of women at high risk of stress. Also, women in this study were mostly White and with high socioeconomic status, which limits our ability to generalize the associations to other populations, for example, among Asian or Black women, or women of other socioeconomic status. Second, misclassification of the exposure by asking women to self-report their perceived stress is possible. However, the PSS-4 is a validated instrument and has been used worldwide to evaluate psychological stress. Third, we have only collected information on stress once during preconception and residual confounding by perceived stress during pregnancy is possible. The main strengths of our study include its prospective assessment and adjustment of important covariates (eg, socioeconomic status) to reduce the concern of confounding, although we do not have data on other important confounders such as sleep or neighborhood environment (eg, prevalence of violent crime) [64] in this cohort study.

In conclusion, we found that maternal preconception psychological stress was positively associated with glucose levels in response to GLT during pregnancy. We also observed that this association was stronger among women conceiving using IUI as well as those who belong to a high socioeconomic status. Given the scarce literature on preconception stress and pregnancy glucose levels, additional studies are needed to corroborate these findings.

## Data Availability

The data are not publicly available due to privacy and confidentiality reasons.

## Abbreviations

BMI: body mass index
EARTH: Environment and Reproductive Health
GDM: gestational diabetes mellitus
GLT: glucose load test
HPA: Hypothalamic-pituitary-adrenal
IQR: interquartile range
ICSI: intracytoplasmic sperm injection
IUI: intrauterine insemination
IVF: in vitro fertilization
PSS-4: Perceived Stress Scale
RR: risk ratio
